# Practical Prediction of Ten Common *Streptococcus pneumoniae* Serotypes/Serogroups in One PCR Reaction by Multiplex Ligation-Dependent Probe Amplification and Melting Curve (MLPA-MC) Assay in Shenzhen, China

**DOI:** 10.1371/journal.pone.0130664

**Published:** 2015-07-07

**Authors:** Lijuan Wu, Xiaomao Yin, Lei Zheng, Jianhua Zou, Ping Jin, Yanwei Hu, Timothy Kudinha, Fanrong Kong, Xu Chen, Qian Wang

**Affiliations:** 1 Laboratory Medical Centre, Nanfang Hospital, Southern Medical University, Guangzhou, Guangdong Province, China; 2 Clinical Laboratory, Bao’an Maternity and Child Health Hospital, Shenzhen, Guangdong Province, China; 3 Clinical Laboratory, The Fifth Affiliated Hospital of Southern Medical University, Guangzhou, Guangdong Province, China; 4 Charles Sturt University, Leeds Parade, Orange, New South Wales, Australia; 5 Centre for Infectious Diseases and Microbiology, Institute of Clinical Pathology and Medical Research–Pathology West, Westmead Hospital, New South Wales, Australia; Instituto Butantan, BRAZIL

## Abstract

**Background:**

*Streptococcus pneumoniae* has more than 95 distinct serotypes described to date. However, only certain serotypes are more likely to cause pneumococcal diseases. Thus serotype surveillance is important for vaccine formula design as well as in post-vaccine serotype shift monitor. The goal of this study was to develop a practical screening assay for ten Shenzhen China common pneumococcal serotypes/serogroups in one molecular reaction.

**Methods:**

A molecular assay, based on multiplex ligation-dependent probe amplification (MLPA) and melting curve (MC) analysis, was developed in an integrated approach (MLPA-MC) for the detection of ten capsular serotypes/serogroups 4, 6 (6A/6B/6C/6D), 9V/9A, 14, 15F/15A, 15B/15C, 18 (18F/18A/18B/18C), 19F, 19A and 23F. We designed serotype/serogroup-specific MLPA probes and fluorescent detection probes to discriminate the different serotypes/serogroups in one molecular reaction. The three steps of MLPA-MC assay are continuous reactions in one well detected by LightCycler 480. A total of 210 *S*. *pneumoniae* isolates from our local Maternity and Child Health Hospital were randomly chosen to evaluate the assay against published multiplex PCR assays.

**Results:**

Our results showed that 198 (94.3%) of *S*. *pneumoniae* isolates were type-able by our assays and the results were in complete concordance with the published multiplex PCRs. Using the MLPA-MC assay, 96 *S*. *pneumoniae* isolates could be typed within 3 hours with limited hands-on time. This serotype/serogroup-screening assay can be easily modified or extended by modification of the serotype/serogroup-specific MLPA probes combinations according to the needs of different laboratories.

**Conclusions:**

We recommend use of this assay as a starting point for screening serotype/serogroup frequencies. There is a need for this assay to be combined with other molecular typing assays, like published serotype specific PCRs, or even the Quellung reaction for serotype confirmation.

## Introduction

*Streptococcus pneumoniae* accounts for about 1 million children deaths annually due to pneumonia and meningitis, mostly in developing countries [1]. The capsular polysaccharide represents an important virulence factor and characterizes *S*. *pneumoniae* into 95 distinct serotypes (within 46 serogroups). There are ten common serotypes/serogroups that account for most paediatric pneumococcal infections worldwide, with serogroups 6, 14, 19 and 23, the most common [2–5]. However, the distribution of serotypes can vary with age, geography and time [2, 6–9]. In China, two nation-wide studies on the distribution of *S*. *pneumoniae* serotypes in invasive pneumococcal diseases (IPD) and in children with pneumonia, showed that 19F, 19A, 23F, 6B and 14 were the most common serotypes, totally accounting for 73.1% of IPD and 87.9% of children with pneumonia [10, 11]. A study in Shenzhen city showed that these five serotypes (19F, 19A, 23F, 6B and 14), accounted for 81.6% of IPD in children[12].

The Quellung reaction or Neufeld test (conventional serotyping) is the gold standard for serotyping, but it’s too expensive for most developing countries including China [9, 10, 13, 14]. In addition, it’s labor-intensive and the interpretation of results is subjective and need expert training. Several different molecular assays have been developed as alternatives to serotyping, with most assays based on serotype-specific or serotype-associated sequences in the *cps* gene cluster, namely *wzy* and *wzx*, or *cpsA-cpsB* (*wzg-wzh*) [9, 15–17].

USA CDC have developed and published a sequential multiplex PCR scheme based on serotype specific PCRs to detect specific pneumococcal serotypes from isolates and directly from clinical specimens, which has been widely used [9]. Multiplex PCR-based methods offer a simple, flexible and economical approach for the surveillance of pneumococcal diseases, and also have the capacity to detect multiple serotypes in mixed serotype specimens. However, these developed schemes need sequential multiplex PCRs, and also gel electrophoresis for identifying PCR products [13, 18]. A single molecular reaction assay, which is rapid, easy and high-throughput and without a need for gel electrophoresis, is preferred for the many busy clinical diagnostic labs.

The multiplex ligation-dependent probe amplification (MLPA) technology uses a simple-to-perform multiplex PCR method, which is able to amplify up to 40 different targets simultaneously [19]. So far, MLPA has been used to detect infectious disease agents, specifically the differentiation of 15 respiratory viruses [20], but has not been developed to identify bacterial (in particular *S*. *pneumoniae*) serotypes. Based on the MLPA technology, and the product identification accomplished by the melting curve (MC) technology, it is highly possible that the different serotypes can be identified by unique melting peak analysis after MLPA reaction in one single well.

The aim of this study was to develop a practical screening assay for ten commonest pneumococcal serotypes/serogroups, in Shenzhen China, in one PCR reaction, as a show case to highlight the potential utility of MLPA-MC technologies in molecular diagnostics. The study targeted the most common five serotypes in Shenzhen China, three PCV7 vaccine serotypes/serogroups (4, 9V/9A and 18C), and the emerging serotypes 15B/15C and 15F/15A[21], to develop a multi-parameter molecular assay for screening serotypes of *S*. *pneumoniae* and evaluated the assay on a set of paediatric clinical isolates.

## Materials and Methods

### Ethics Statement

The study protocol was approved by the Human Research Ethics Committee of Bao’an Maternity and Child Health Hospital (No. S-2013002), and written consent was obtained from the next of kin, caretakers, or guardians on behalf of the children enrolled in this study.

### *S. pneumoniae* Isolates

Thirty-nine reference *S*. *pneumoniae* isolates, representing different serotypes, were used as a control set to test MLPA-MC specificity and to determine cross-reactivity. They included serotypes 4, 14, 6A, 6B, 6C, 6D, 9V, 9A, 15F, 15A, 15B, 15C, 18F, 18A, 18B, 18C, 19F, 19A, and 23F, which were related to the target serotypes/serogroups in this study. Other twenty serotypes (1, 2, 3, 5, 8, 20, 7F, 7C, 9N, 9L, 10A, 11A, 11D, 12F, 17F, 22F, 22A, 23A, 23B and 33F) that were not targeted in this study, were also included in but as negative controls. In addition, 5 to 10 known serotype isolates for each of the ten serotypes/serogroups targeted in this study were used to access the probe sets. Finally, 210 additional pneumococcal isolates obtained from children, including 30 invasive pneumococcal isolates (Jan 2009 to Jan 2012), and 180 induced sputum source pneumococcal isolates (Jan 2012 to June 2012), from Bao’an Maternity and Child Health Hospital, Shenzhen, China, were chosen for a double-blinded study to evaluate the performance of the MLPA-MC assay against 3 multiplex PCRs selected from 8 USA CDC sequential multiplex PCR assays which target the ten serotypes/serogroups in the study (http://www.cdc.gov/streplab/pcr.html). All the 210 pneumococcal isolates were confirmed using standard microbiological tests, including colony morphology, optochin susceptibility and bile solubility.

### DNA Extraction from Bacterial Isolates

Pneumococcal isolates were retrieved from storage by subculture on blood agar plates and incubated overnight at 35°C in 5% CO_2_. Genomic DNA was extracted from bacteria by using the AxyGenamp DNA Mini Extraction Kit (Axygen, USA). Extraction was performed according to the manufacturer’s instructions and the purified nucleic acid was diluted in a final volume of 100 μL Tris EDTA buffer and stored at -20°C until use.

### MLPA Combined with Melting Curve Analysis (MLPA-MC) Probes Design

**Fig 1:**
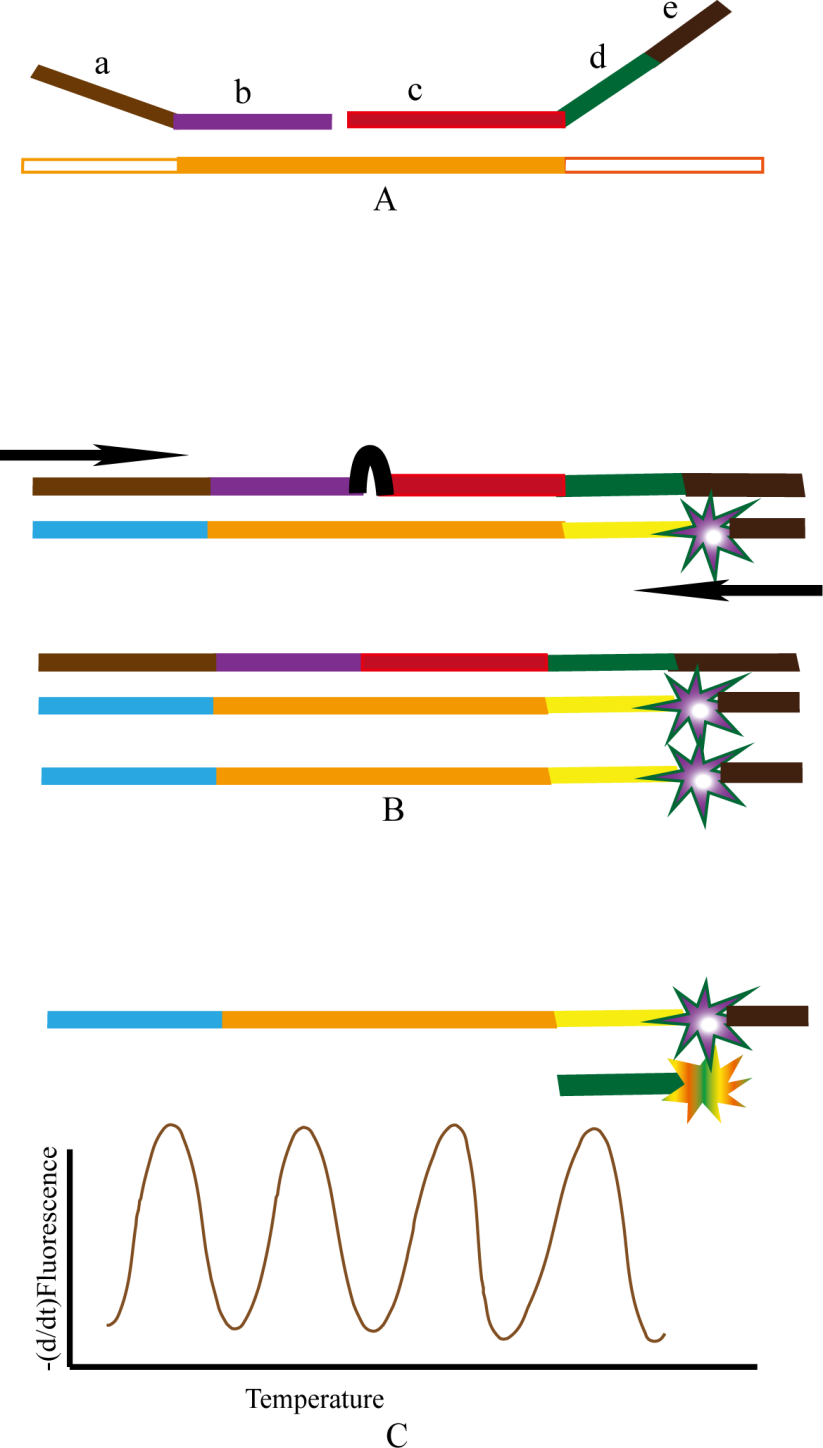
Overview of the MLPA-MC assay. (A) Hybridization: A pair of MLPA probe consists of left and right probe: (a) Universal forward primer binding site (5’-GGGTTCCCTAAGGGTTGGA-3’) will be the same to the universal forward primer (5’-GGGTTCCCTAAGGGTTGGA-3’), (b) Left probe oligonucleotides (LPO): Left serotype specific hybridization regions; (c) Right probe oligonucleotides (RPO): Right serotype specific hybridization regions, (d) Serotype specific stuffer sequence, (e) Universal reverse primer binding site (5’-TCTAGATTGGATCTTGCTGGCAC-3’) will be targeted by universal reverse primer, 5’-GTGCCAGCAAGATCCAAT-(labeled FAM)CTAGA-3’. The LPO and RPO hybridize specifically to the target DNA sequences adjacent to each other. (B) Ligation and PCR: LPO and RPO joined by ligation enzyme. The linked MLPA probes are amplified by an asymmetric PCR reaction by one universal primer set. The PCR products consist of ds-DNA and ss-DNA.(C) MC analysis: Melting curve assay of the hybridized specific fluorescent detection probe and the ss-DNA of PCR products.

The MLPA probes were designed as described earlier[19]. Twelve pairs of MLPA probes were designed and included ten pairs of serotype/serogroup-specific (4, 6, 9V/9A, 14, 15A/15F, 15B/15C, 18, 19A, 19F and 23F) probes and two pairs of internal controls (*cpsA* and *lytA*). The internal controls were used as “catch-all” controls, as both are species-specific and found in almost all *S*. *pneumoniae*, except non-capsular isolates that lack *cpsA*. MLPA probes contained left and right probes. The left probe consisted of: (a) the universal forward primer binding site, and (b) serotype/serogroup specific left probe oligonucleotides (LPO); the right probe consisted of: (c) serotype/serogroup-specific right probe oligonucleotides (RPO), (d) the serotype/serogroup-specific stuffer sequence, and (e) the universal reverse primer binding site. Among them, the LPO (b) and RPO (c) hybridize specifically to template DNA. We specifically designed them against conserved gene within each serotype/serogroup, but with heterogeneity between targeted and the rest of the serotypes/serogroups. The specificities of LPO and RPO were checked and determines by blastn the serotype specific PCR products against GenBank database (http://www.ncbi.nlm.nih.gov), with *T*m near 70°C and without SNP. Each stuffer sequence (d) determined the specific detection of product, while the universal forward (a) and reverse (e) primer binding site of each MLPA probe is the same (forward primer binding sequence, 5’-GGGTTCCCTAAGGGTTGGA-3; reverse primer binding sequence, 5’-TCTAGATTGGATCTTGCTGGCAC-3). The MLPA probe design is shown in Fig 1A.

The serotype/serogroup-specific fluorescent detection probe had the same sequence as the specific MLPA stuffer sequence, and each sequence, whose source was bacteriophage, was from the commercial respiratory virus diagnostic kit made by Pathofinder (http://www.pathofinder.com/; The Netherland) with some minor modifications [20]. The twelve probes were labelled with ROX or CY5 fluorescence. Each probe had a specific *Tm* for melting curve assay which was used as a marker to discriminate the different serotypes. It could be used repeatedly in different serotypes/serogroups panels. All the probes were synthesized by Invitrogen (Shanghai, China). The probes details are shown in Table 1.

**Table 1:** The Probes Details of the MLPA-MC Assay. ROX, rhodaminein X; Cy5,Cascade yellow 5;

serogroup	MLPA probe						Fluorescent detection probe		
	Target gene	GenBank no	start	Sequence of LPO	Sequence of RPO	Product size (bp)	Sequence	Dye	Tm
**4**	*wzy*	CR931635	9341	GGTCACATCTACTTCTTGCGTCACTGTATCTGA	CGATAAAATTTGTAATCCCGATGCCTGAGCCTC	135	GGCACAGCGATTGCGTTGAGGAGTCCG	ROX	**75**
**6**	*wci*P	CR931639	8806	GAGTATGGGAAGGTGTTGTTCTGCCCT	GAGCAACTGGTCTTGTATCGAAGACATGGAC	119	TCCGTCCTTAGAGTCCGCT	ROX	**65**
**9V/9A**	*wzy*	CR931648	10676	CCGTTCGAGCAAGTATATCTAGTAAGGTATTGTGT	GCGGAGTTAACGATAATCCCATTTGTCCAAGCA	127	TCTCCACAGGTAAATCT	Cy5	**55**
**14**	*wzy*	CR931662	8002	TCTACTGTAGAGGGAATTCTGACACCT	GCGCCAAGTAACATTTCCATTCCA	109	ACTAGGAGAGTGGTCA	ROX	**55**
**18**	*wzy*	CR931673	11488	GTGGGTTGTTTTAGTGATTACAATGGGCTGTGC	TTTCGGTTTAGCAGGAGTTTCTGCAACCTTTGC	127	AGCCAGAGTGGTCTTAATG	Cy5	**60**
**19F**	*wzy*	CR931678	11298	CGAGTTATGAAGGTGAATTGACAGTGCGAACTTTT	ATTCGAGTTCTCATTCGTGTTATTGACGTATCTGC	130	CATGCCTAATGGTCCAGT	ROX	**60**
**19A**	*wzy*	CR931675	9672	CTACCAGTTATGAAGGTGAGCTAACAGTGCGAA	CTTCGATTCGGGTCCTCATTCGTATCATTGACG	126	GGTCTTCGCCCAGAAGCT	Cy5	**65**
**23F**	*wzy*	CR931685	9085	TAACGCCAGTAAAGAGATGACTATTGTCGT	TGCTGTCACTACCAGTCTATGCTCTT	123	CAGGTCGTTACGTGGATTAGCGGTC	ROX	**70**
**15F/15A**	*wzy*	CR931663	7971	GTCCCATAGGAAGGAAATAGTATTTGTTCGTCC	CGCAAACTCTGTCCTATTCTCATATGATAATGCC	134	ACGGATGCAATAGAACTCTTCGCGC	Cy5	**70**
**15B/15C**	*wzy*	CR931665	7531	GGAAGAAGCTTATTAGGTTGGGACGGATTCGTA	TCAGCTACCAGTTACGGAGTAAGATATGCAGGA	135	GCCGTGCTGACCGTTCTCGTATGTGCG	Cy5	**75**
***cps*A**	*wzg*	CR931662	1459	CTGTGTCGCTCTTTGCAGTACAGCAGTT	TGTTGGACTGACCAATCGTTTAAATGCGAC	136	TGAGGCACTTGCTGGGACTCCACAGACCCAGTGCTG	Cy5	**80**
***lyt*A**	*lyt*A	AM113493	842	CAGTGTTCCGTCTGGTTTGAGGTAGTACC	AACCTGTTCCGTCCGCTGACTGGATAAA	136	CTCAGCTGAGTCCGCTCCGACAGCAGGCACTATATTC	ROX	**80**

### MLPA-MC Reaction

MLPA reaction was performed essentially as described earlier [19], with some modifications.The three steps of MLPA-MC assay are continuous reactions in one well detected by LightCycler 480(Roche): A.Hybridization. The template DNA hybridize to specific MLPA probes. The mixture included 5 μL *S*. *pneumoniae* DNA sample, 1.5 μL probe mixture (0.5 fmol of each serotype specific MLPA probe in TE buffer; included all the 10 targeted serotypes/groups and *cpsA* and *lytA* probes), and 1.5 μL MLPA buffer (1.5 M KCl, 300 mM Tris-HCl, pH 8.5, 1 mM EDTA) in one well (8 μl total sample volume). Denaturation was performed for 10 min at 98°C and then the mixture incubated for 1h at 60°C for hybridization. B.Ligation and PCR. The hybridized mixture was subsequently diluted to 40 μL by addition of 32 μl ligation and PCR buffer. The 32 μL ligation and PCR reaction buffer contained the following: 2.5 mM MgCl_2_, 10 mM Tris-HCl, 0.2 mM Nicotinamide Adenine Dinucleotide (NAD), deoxynucleoside triphosphates 0.2 mM each, 1 U *Taq* polymerase, 1 U heat-stable ligase 65 enzyme, 12 pmol fluorescent detection probes (12 probes included, 1 pmol each), 0.3 μM of the universal PCR primers (forward primer, 5’-GGGTTCCCTAAGGGTTGGA-3; reverse primer, 5’-GTGCCAGCAAGATCCAAT-(labeled FAM)CTAGA-3’. The ratio of forward and reverse primer was 1:2. FAM, Carboxyfluorescein). And by incubating 15 min at 54°C for ligation, the hybridized LPO and RPO are joined by ligase to an integral MLPA probe, followed by PCR amplification. The integral MLPA probes were amplified by an asymmetric PCR reaction with one universal primer set. The PCR amplification was as follows: an initial cycle of 2 min at 95°C, followed by 10 cycles of 30 s at 94°C, 30 s at 60°C, and 1 min at 72°C and 23 cycles of 30 s at 94°C, 30 s at 50°C, and 1 min at 72°C. The PCR products were ds-DNA and ss-DNA labeled with FAM.C. Melting Curve analysis (MC).Melting Curve analysis assay (MC) of the hybridized specific fluorescent detection probe and the ss-DNA of PCR products was performed as follows: Firstly, an initial denaturing step of 2 min at 95°C was carried out, which eliminated any non-specific binding, and then followed the specific binding of fluorescent detection probe and the ss-DNA labeled with FAM. The binding illuminates the ROX/CY5 according to fluorescence resonance energy transfer (FRET) and the melting peak is determined by the (d/dt) fluorescence from 45°C~85°C. The overall MC analysis time is about ten minutes. The overview of MLPA-MC is shown in Fig 1. All buffers and enzymes for MLPA-MC were obtained from Invitrogen (Shanghai, China). All clinical isolates (*n* = 210) were analyzed by MLPA-MC assay.

### Sensitivity, Specificity and Reproducibility of MLPA-MC

**Fig 2:**
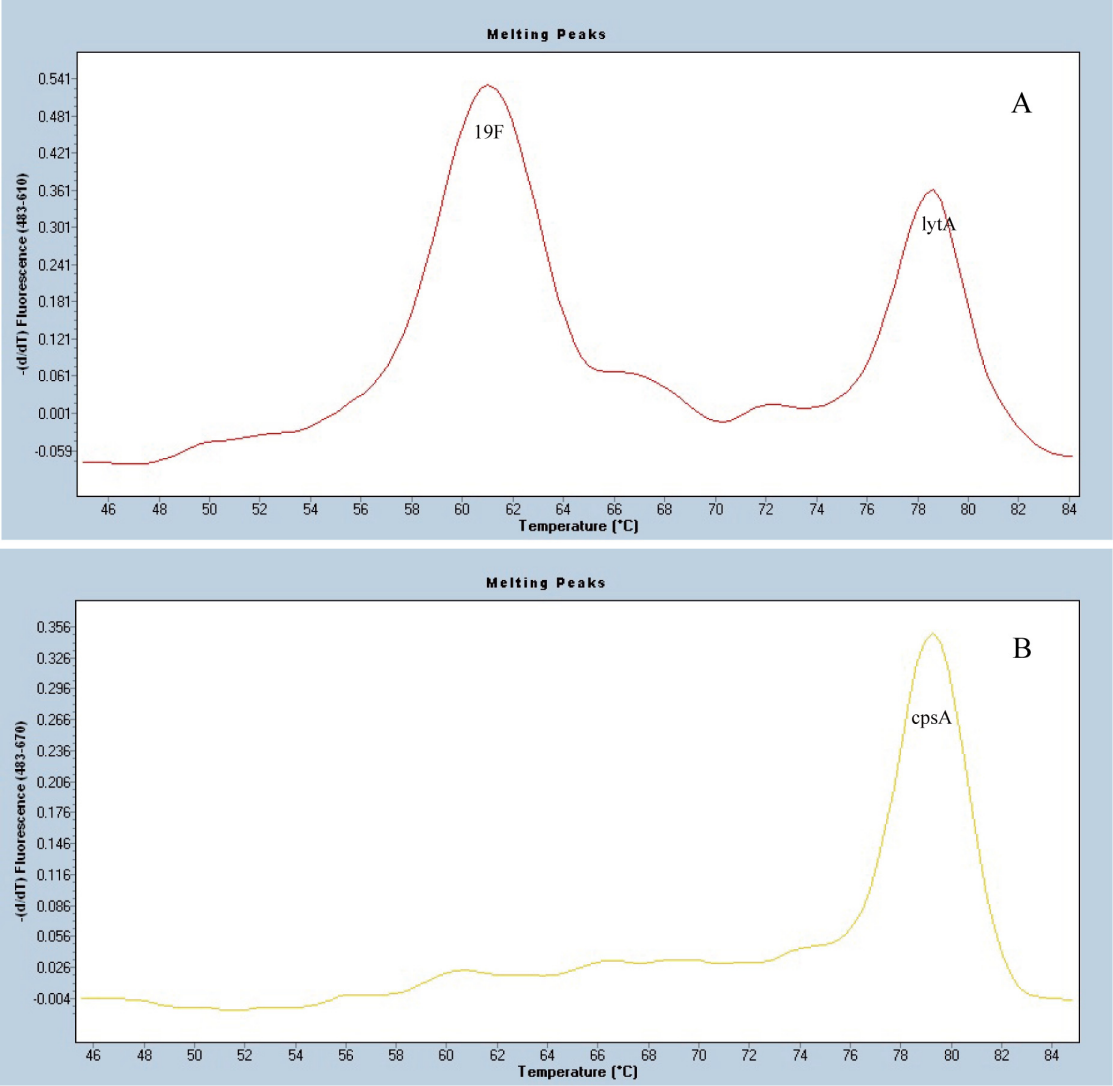
Melting curve analysis of 19F control isolate. (A) 19F serotype specific melting peak and internal control *lytA* peak (ROX dye).(B) Melting peak of the internal control *cpsA* (Cy5 dye).

**Fig 3:**
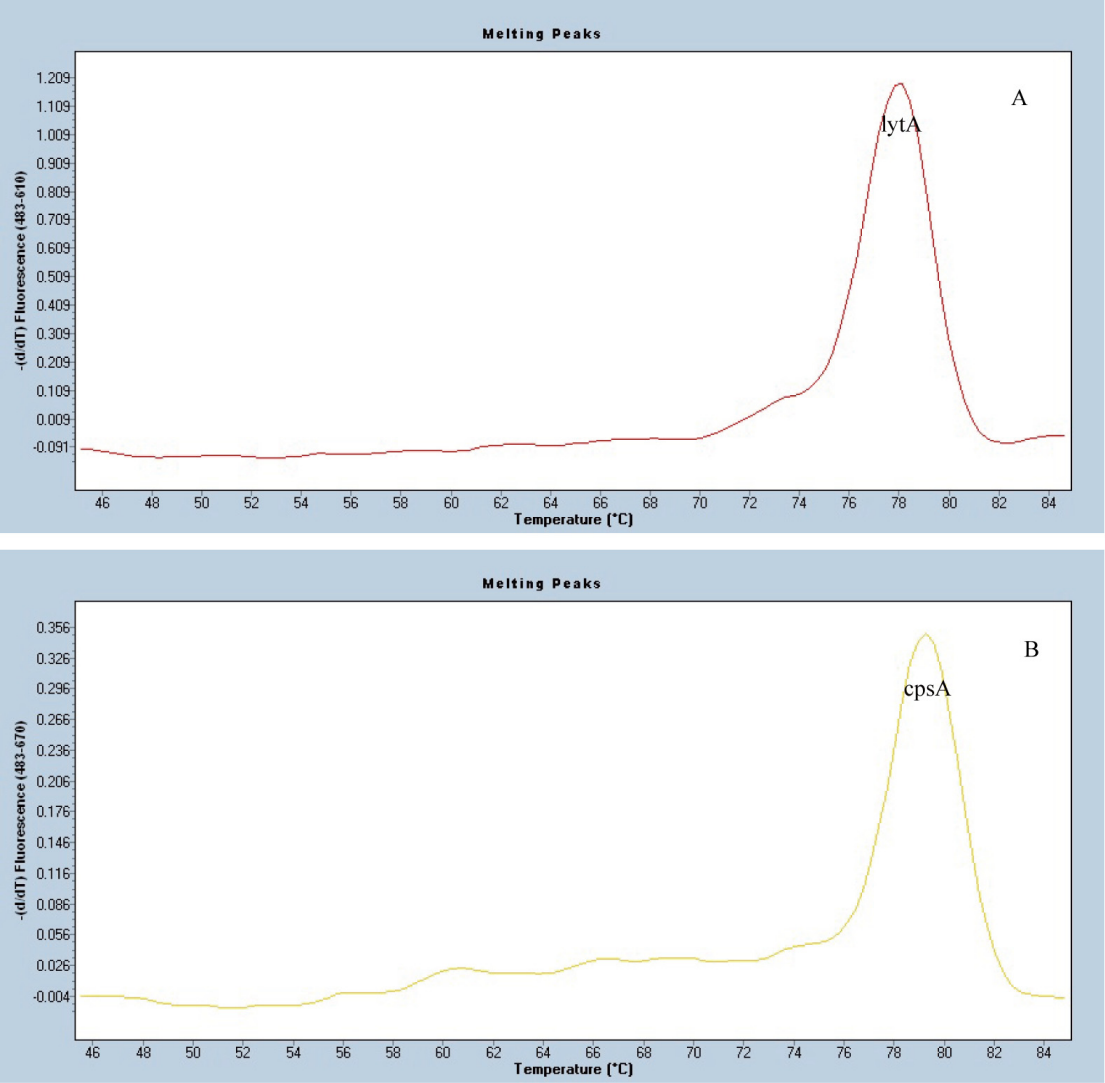
Melting curve analysis of one negative control isolate. (A) Melting peak of the internal control *lytA* (ROX dye). (B) Melting peak of the internal control *cpsA* (Cy5 dye).

**Fig 4:**
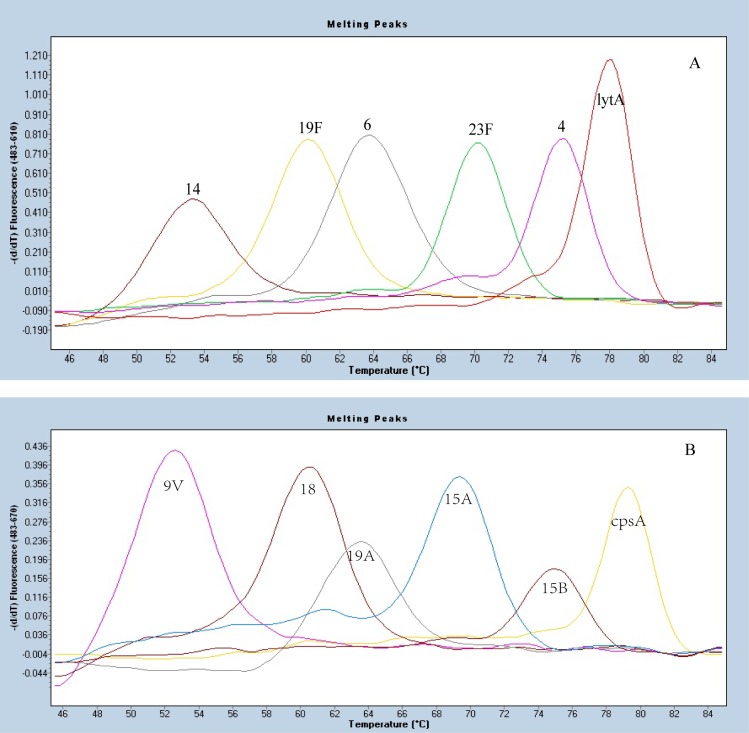
Melting curve analysis of all 12 different fluorescent detection probes. (A) Melting peaks of five serotype/serogroup-specific fluorescent detection probes 14, 19F, 6, 23F, 4 and internal control *lytA* probe (ROX dye). (B) Melting peaks of the other five serotype/serogroup-specific fluorescent detection probes 9V, 18, 19A, 15F/15A, 15B/15C, and internal control *cpsA* probe (Cy5 dye).

Sensitivity of MLPA-MC assay was tested on serial dilutions of cloned DNA standards with a minimal threshold for detection set at 1000 copies for each probe in the panel. Serotype/serogroup-specificities of the specific probes were accessed by testing the control isolates. When the target control isolates were tested by MLPA-MC assay, the results showed three melting peaks, the serotype/serogroup specific peak, the *cpsA*, and *lytA* peaks, as shown in Fig 2. For the other serotypes/serogroups (other than the 10 targeted serotypes/serogroups) isolates that were used as negative or specificity controls, only *cpsA* and *lytA* peaks were obtained, as in Fig 3. The actual melting peak of each pair of probes fluctuated in the range of ±0~2 centigrade (°C) to the expected *Tm*, which was acceptable, as shown in Fig 4. No cross-reactivity among the ten target probes was observed when they were tested against the control set. Of the ten probes, five were specific for the target serotypes of 4, 14, 19F, 19A and 23F. For USA CDC specific mPCRs, the PCR primers can’t discriminate between serotypes 9V and 9A, 15B and 15C, 15F and 15A, four serotypes of serogroup 18 (18F/18A/18B/18C) and four serotypes of serogroup 6 (6A/6B/6C/6D), respectively. In this study, serotype 9V/9A probe reacted with both serotypes 9V and 9A; serotype 15F/15A probe reacted with both serotypes 15F and 15A; serotype 15B/15C probe reacted with both serotypes 15B and 15C; serogroup 18 probe can react with all 4 serotypes of 18F/18A/18B/18C; serogroup 6 probe can react with all 4 serotypes of 6A/6B/6C/6D. Reproducibility of our results depended on the purity of DNA samples and the thoroughness of mixing during the reactions.

### Multiplex PCR Reaction

Ten USA CDC mPCR primer pairs were used to target serotypes/serogroups in this study. The sequences for the serotype(s)-specific primers, the PCR conditions and product detections are available at the USA CDC web page (http://www.cdc.gv/ncidod/biotech/strep/pcr.htm), which have also been described elsewhere[13, 18]. The primers were grouped into three multiplex reactions as below: reaction one included three primer pairs for serotype/serogroups 4, 6, and 18; reaction two included four primer pairs for serotype(s) 14, 9V/9A, 15F/15A, and 19A; and finally reaction three included three primer pairs for serotype(s) 15B/15C, 19F, and 23F. Additionally, a primer pair (primer *cpsA*-*f* and *cpsA*-*r*) was included in each reaction as an internal control. Each serotype in one reaction can be distinguished by a specific product size. The representative mPCR reactions are shown in S1 Fig.

All primers were synthesized by Invitrogen (Shanghai, China), whilst all PCR related enzymes, and other reagents were from Takara Bio (Dalian, China). All the 210 *S*. *pneumoniae* isolates were analyzed by the three multiplex PCR reactions.

## Results

### MLPA-MC Analysis of the Clinical Isolates

For the 210 clinical isolates, 198 (94.3%) isolates were clearly discriminated by one reaction MLPA-MC assay, and the actual melting peaks of each serotype fluctuated in the range of ±0~2 centigrade (°C) to the expected *Tm*. The isolates were discriminated into the following serotypes/serogroups; 19F (n = 67, 31.9%), 23F (n = 40, 19.1%), 19A (n = 22, 10.5%), 6 (n = 37, 17.6%), 15B/15C (n = 14, 6.7%), 14 (n = 13, 6.2%), 15A/15F (n = 2, 1.0%), 4 (n = 1, 0.5%), 18 (n = 1, 0.5%) and 9V/9A (n = 1, 0.5%). Twelve isolates (5.7%) were non-typeable by MLPA-MC assay. Further results are shown in Table 2.

**Table 2:** Serotype Results Identified by MLPA-MC for 210 Pneumococcal Isolates.

Serotype results	Positive isolates		Actual MC Results	
	no	%	Tm	Dye
**19F**	67	31.9	61±1	ROX
**23F**	40	19.1	71±1	ROX
**19A**	22	10.5	64±1	CY5
**6**	37	17.6	64±1	ROX
**15B/15C**	14	6.7	75±1	CY5
**14**	13	6.2	54±1	ROX
**15F/15A**	2	1.0	69	CY5
**4**	1	0.5	75	ROX
**18**	1	0.5	60	CY5
**9V/9A**	1	0.5	53	CY5
***cpsA***	209	99.5	79±1	CY5
***lytA***	210	100	79±1	ROX
**Non-typeable in this study**	12	5.7	no serotype specific melting peak	

### MLPA-MC Versus Multiplex PCR Reaction

The MLPA-MC assay identified the same serotype or group as that identified by mPCR in all 198 clinical isolates. Both MLPA-MC and mPCR failed to identify the serotypes of 12 isolates. Thus agreement of the MLPA-MC and mPCR was 100% for the clinical isolates. However, the MLPA-MC assay had a high throughput, with a batch of 96 reactions carried out in 3 hours. Consequently, the 210 MLPA-MC reactions were performed in 9 hours, with limited hands-on time. On the other hand, at least 400 mPCR reactions performed according to the mPCR scheme needed a total of 18 h of work. Besides PCR instrument, other additional requirements for the mPCR test included electrophoresis, Gel Imager and also needed more hands-on time. The comparison details are shown in Table 3.

**Table 3:** Comparison of MLPA-MC to mPCR reaction.

	mPCR	MLPA-MC
**Target gene**	Serotype specific *cps* gene	Serotype specific *cps* gene
**PCR amplified template**	Target DNA	Specific MLPA probe
**PCR primer needed**	Multiplex serotype specific primers	A pair of universal primers
**Discrimination of serotype by**	Serotype specific primers	Serotype specific MLPA probes, serotype specific fluorescent detection probes
**Reaction steps**	Multiplex PCR, electrophoresis, gel imaging, separately	Continuous reaction in one well: hybridization, ligation and PCR, melting curve analysis
**PCR products size & type &detection by**	160~753 bp; dsDNA & electrophoresis	109~136 bp; dsDNA, ssDNA; melting curve assay
**Turn around time**	~ 9 h	~3 h
**Analytic machines**	Thermal cycler; electrophoresis; Gel Imager	LightCycler 480 or other fluorescence reader

## Discussion

To our knowledge, this is the first study to determine the ten common serotypes/serogroups of *S*. *pneumoniae* using MLPA-MC assay in one PCR reaction. Both the serotype-specific MLPA probes and fluorescent detecting probes were the key of the MLPA-MC assay that determined the specificity of the assay. Our study indicates that *cps* serotype/serogroup-specific genes can also be targeted to efficiently and reliably determine serotype specific MLPA probes besides the PCR primers [9, 14, 17, 22, 23]. Compared to serotype specific PCR for the amplification of the target template DNA, the MLPA assay amplifies the MLPA probe itself [19]. The concentration of each serotype specific MLPA probe in the hybridization system is 10^7^ pmol/L, which is much higher than that of template DNA. The hybridization time is 1 hour in the system, which is enough for the bacteria genome though much longer (less than 16 h)is needed for human genome. The combination of the MC with MLPA for identification of the different products, was a modification of the traditional MLPA assay that made the assay more automatic, and simple for busy clinical diagnostic labs. As Table 3 shows, the MLPA-MC assay has advantages of both the mPCR and the real time PCR, with high throughput and continuous reactions in one well. The mPCRs combined with additional step of detection of products like gel electrophoresis, capillary electrophoresis, and reverse line blot microarrays have been used effectively in different studies [9, 13, 16–18, 24]. Real time PCR is more sensitive and automatic than mPCR for diagnosis and serotyping in children with culture negative pneumococcal invasive disease, but is limited by throughput [25].

The MLPA-MC assay successfully assigned 198 (94.3%) of the clinical isolates to the correct serotype/serogroup in total agreement to those from the mPCR reactions. The most common five serotypes (19F, 23F, 19A, 6, and 14) accounted for 85.2% of the isolates, which is in agreement to another study in China [12]. However, in the present study, the prevalence of serotype15B/15C was unexpectedly high, accounting for 6.7% of the isolates, which was different from other studies [10–12], suggesting that 15B/15C is a common serotype in Shenzhen China and should be included in the screening test.

Our study has a limitation in that it could not determine the serotypes within serogroup 6, so we proposed using MLPA-MC as serogroup 6 screening assay, in line with USA CDC mPCR assays. Thereafter, published serotypes 6A-6D serotype-specific PCR would be used for further differentiation within serogroup 6 [15, 26, 27]. We have completed serotypes 6A/6B/6C/6D-specific PCR and the frequencies of serotypes 6A, 6B and 6C among 27 serogroup 6 isolates in the study were 44.4% (12/27), 48.1% (13/27) and 7.4% (2/27), respectively; serotype 6D was not found in the isolates. As the GenBank and PubMed analysis suggested that new serotypes 6E and 6F in serogroup 6 were still coming out, we intend to design a new MLPA-MC panel that focuses on serogroup 6 in the future when the serotypes in serogroup 6 are clearer.

In this study, we provide only one serotype/serogroup screening panel by using of MLPA-MC assay, but it can be easily adapted to suit other panels of serotypes. The whole reaction system can be easily repeated in different panels. Only modification of the LPO and RPO to target the new serotype/serogroup-specific *cps* sequence is required to complete design of a new serotype specific MLPA probe. Fluorescent detecting probes can be used repeatedly in different serotype panels. We have designed another 5 serotype specific MLPA probes for serotypes1, 2, 3, 5, 7F/7A and listed as S1 Table. which would be a new panel, but need further comprehensive evaluation before clinical use. Later we plan to design more serotype specific MLPA probes to extend the system, with more MLPA-MC panels to be chosen.

We recommend using this MLPA-MC assay as a starting point for screening serotype/serogroup frequencies. This assay can be combined with other molecular typing assays or even the Quellung reaction for serotype confirmation, like for serotypes in serogroup 6. It can also be used for serotype determination in pure culture isolates as part of serotype surveillance studies before and after introduction of pneumococcal vaccination. By using the popular LightCycler 480, the MLPA-MC assay can be easily introduced to most clinical labs and readily assign results to 96 isolates in half a day with high throughput and little manual labour.

## Supporting Information

S1 Fig Representative multiplex PCR products patterns. (A) mPCR reaction one–for serotype 4, serogroup 6 and serogroup 18. (B) mPCR reaction two–for serotype 14, serotypes 9V/9A, serotypes 15F/15A and serotype 19A. (C) mPCR reaction three–for serotype 15B/15C, serotype 19F and serotype 23F.
(TIF)

S1 Table The New Probes Details of the MLPA-MC Assay. (DOC)
