# Supplementary figures and images for: Practical Prediction of Ten Common Streptococcus pneumoniae Serotypes/Serogroups in One PCR Reaction by Multiplex Ligation-Dependent Probe Amplification and Melting Curve (MLPA-MC) Assay in Shenzhen, China
Source: PLoS One. 2015 Jul 7;10(7):e0130664. doi: 10.1371/journal.pone.0130664 (PMC4495002; doi:10.1371/journal.pone.0130664)

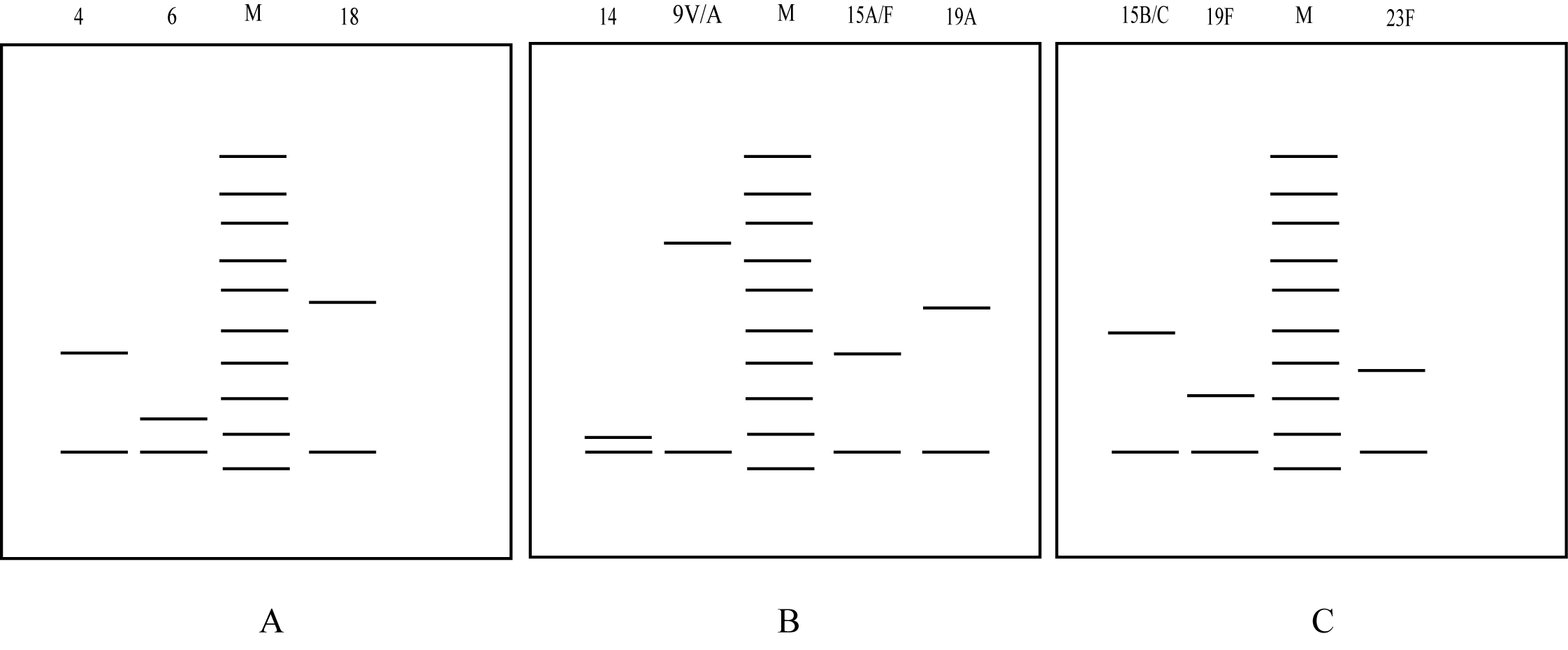

Supplement: S1 Fig — (A) mPCR reaction one–for serotype 4, serogroup 6 and serogroup 18. (B) mPCR reaction two–for serotype 14, serotypes 9V/9A, serotypes 15F/15A and serotype 19A. (C) mPCR reaction three–for serotype 15B/15C, serotype 19F and serotype 23F. (TIF) [file pone.0130664.s001.tif]
