# Supplementary material for: Practical Prediction of Ten Common Streptococcus pneumoniae Serotypes/Serogroups in One PCR Reaction by Multiplex Ligation-Dependent Probe Amplification and Melting Curve (MLPA-MC) Assay in Shenzhen, China
Source: PLoS One. 2015 Jul 7;10(7):e0130664. doi: 10.1371/journal.pone.0130664 (PMC4495002; doi:10.1371/journal.pone.0130664)
Supplement: S1 Table — (DOC) [file pone.0130664.s002.doc]

**S1.Table. The New Probes Details of the MLPA-MC Assay.**

| **serotype** | **MLPA probe** | | | | | | **Fluorescent detection probe** | | |
| --- | --- | --- | --- | --- | --- | --- | --- | --- | --- |
|  | **Target gene** | **GenBank no** | **start** | **Sequence of LPO** | **Sequence of RPO** | **Product size (bp)** | **Sequence** | **Dye** | **Tm** |
| **1** | *wzy* | CR931632 | 10073 | AATAACTATTTATTATTTCTCAATAATATGA | ATACGTTTATCACAACAGTAGCAATTATATA | 120 | ACTAGGAGAGTGGTCA | ROX | 55 |
| **2** | *wzy* | CR931633 | 10291 | CCACTACACCTATTTTGCATATGTTTTGTTACTAGCTT | TTATTTTGGTAATTGTTATCCCATATAAGAACCGAGTG | 136 | CATGCCTAATGGTCCAGT | ROX | 60 |
| **3** | *galU* | CR931634 | 9042 | GGAAAGTAGTAATGGCCTCTATAGTGTTGATGC | TTTTGTAGAGAAACCAAAACCAGAAGAAGCGCC | 127 | TCCGTCCTTAGAGTCCGCT | ROX | 65 |
| **5** | *wzy* | CR931637 | 6433 | TTCACTACTTTCAAGGACATACTTTTTCAAA | TATTGATTATGTTTATCGAGCTAAGAATTCA | 129 | CAGGTCGTTACGTGGATTAGCGGTC | ROX | 70 |
| **7F/7A** | *wzy* | CR931643 | 14932 | TTTACCTCGTTCCAATATTTTGTGCTTTTAA | AATCAGGAGATTTTCTTGGAGGAATTTTTGG | 131 | GGCACAGCGATTGCGTTGAGGAGTCCG | ROX | 75 |
| ***lyt*A** | *lyt*A | AM113493 | 842 | CAGTGTTCCGTCTGGTTTGAGGTAGTACC | AACCTGTTCCGTCCGCTGACTGGATAAA | 136 | CTCAGCTGAGTCCGCTCCGACAGCAGGCACTATATTC | ROX | 80 |

ROX ,rhodaminein X;
